# Genetic heterogeneity of dolphin morbilliviruses detected in the Spanish Mediterranean in inter-epizootic period

**DOI:** 10.1186/s12917-018-1559-0

**Published:** 2018-08-24

**Authors:** Consuelo Rubio-Guerri, M. Ángeles Jiménez, Mar Melero, Josué Díaz-Delgado, Eva Sierra, Manuel Arbelo, Edwige N. Bellière, Jose L. Crespo-Picazo, Daniel García-Párraga, Fernando Esperón, Jose M. Sánchez-Vizcaíno

**Affiliations:** 1 VISAVET Center and Animal Health Department, Veterinary School, Complutense University of Madrid, Avda. Puerta del Hierro s/n, 28040 Madrid, Spain; 2 Fundación Oceanografic de la Comunitat Valenciana, C/. Eduardo Primo Yúfera (Científic) 1B, 46013 Valencia, Spain; 3 Medicine and Surgery Department (Anatomic Pathology), Veterinary School, Complutense University of Madrid, 28040 Madrid, Spain; 4 Unit of Histology and Veterinary Pathology, Institute for Animal Health, Veterinary School, University of Las Palmas de Gran Canaria, Trasmontaña, s, /n 35416 Arucas (Las Palmas), Canary Islands Spain; 5 National Institute for Agricultural and Food Research and Technology, Ctra. de Algete a El Casar s/n, 28130 Madrid, Spain; 6 Veterinary Services, Avanqua Oceanogràfic S.L., C/ Eduardo Primo Yúfera (Científic) 1B, 46013 Valencia, Spain

**Keywords:** Paramyxoviridae, Endemic ocurrence, Dolphin morbillivirus, *Stenella coeruleoalba*, Mediterranean striped dolphin

## Abstract

**Background:**

In the last 20 years, Cetacean Morbillivirus (CeMV) has been responsible for many die-offs in marine mammals worldwide, as clearly exemplified by the three dolphin morbillivirus (DMV) epizootics of 1990–1992, 2006–2008 and 2011 that affected Mediterranean striped dolphins (*Stenella coeruleoalba*). Systemic infection caused by DMV in the Mediterranean has been reported only during these outbreaks.

**Results:**

We report the infection of five striped dolphins (*Stenella coeruleoalba*) stranded on the Spanish Mediterranean coast of Valencia after the last DMV outbreak that ended in 2011. Animal 1 stranded in late 2011 and Animal 2 in 2012. Systemic infection affecting all tissues was found based on histopathology and positive immunohistochemical and polymerase chain reaction positive results. Animal 3 stranded in 2014; molecular and immunohistochemical detection was positive only in the central nervous system. Animals 4 and 5 stranded in 2015, and DMV antigen was found in several tissues. Partial sequences of the DMV phosphoprotein (P), nucleoprotein (N), and hemagglutinin (H) genes were identical for Animals 2, 3, 4, and 5, and were remarkably different from those in Animal 1. The P sequence from Animal 1 was identical to that of the DMV strain that caused the epizootic of 2011 in the Spanish Mediterranean. The corresponding sequence from Animals 2–5 was identical to that from a striped dolphin stranded in 2011 on the Canary Islands and to six dolphins stranded in northeastern Atlantic of the Iberian Peninsula.

**Conclusions:**

These results suggest the existence of an endemic infection cycle among striped dolphins in the Mediterranean that may lead to occasional systemic disease presentations outside epizootic periods. This cycle involves multiple pathogenic viral strains, one of which may have originated in the Atlantic Ocean.

## Background

*Cetacean morbillivirus* (CeMV) is an enveloped, negative-strand RNA virus within the genus *Morbillivirus* and family *Paramyxoviridae* [1] that may cause serious respiratory, lymphoid and central nervous system (CNS) disease in susceptible cetacean species, leading to strandings and death. Three main lineages of CeMV have been described, dolphin morbillivirus (DMV) [2], porpoise morbillivirus (PMV) [1], and pilot whale morbillivirus (PWMV). Dolphin and porpoise morbilliviruses caused mass mortalities in several cetacean species, and pilot whale morbillivirus has been reported sporadically in pilot whales [3–5], although this species has been reported also to be infected by DMV [6]. In addition to these three lineages of CeMVs, three more recent strains have been identified in stranded cetaceans [7–9].

The first recognized morbilliviral epizootic of cetaceans (actually the first reported for any marine mammal) occurred in 1987–88 on the Atlantic coast of the USA, killing an estimated 50% of the regional bottlenose dolphins (*Tursiops truncatus*) [10]. In 1990 approximately 1000 Spanish Mediterranean striped dolphins (*Stenella coeruleoalba*) were killed by a DMV epizootic [11]. At least another 100 CeMV-caused deaths of bottlenose dolphins occurred in 1993 in the Gulf of Mexico [12, 13]. Epizootic episodes occurred again in the Spanish Mediterranean in 2007 and 2011, with over 200 and 50 deaths of striped dolphins, respectively [14–17], caused by two variants of the same DMV strain (judged from viral phosphoprotein sequences).

Since 2011, CNS-restricted morbilliviral infection (MI) was reported in several animals stranded on the Italian Mediterranean coast in 2012 [18–20] and mass mortality of striped dolphins and of sperm whales due to sytemic infection were recorded in Italy in 2013 and 2016, respectively [21, 22]. Although isolated cases of systemic infection (several tissues affected) and CNS-restricted infection have been reported in dolphins in the Atlantic Ocean and in Italy in a fin whale [2, 23–25], no systemic infection has been reported in dolphins in the Spanish Mediterranean outside mass outbreaks. Here we report some cases of systemic DMV infection in striped dolphins in the Spanish Mediterranean in the 2011–2016 period, exploiting sequence comparisons of DMV phosphoprotein (P) sequences to suggest potential Atlantic origins for the cases detected from 2012 till now.

## Methods

### Animals

**Fig. 1:**
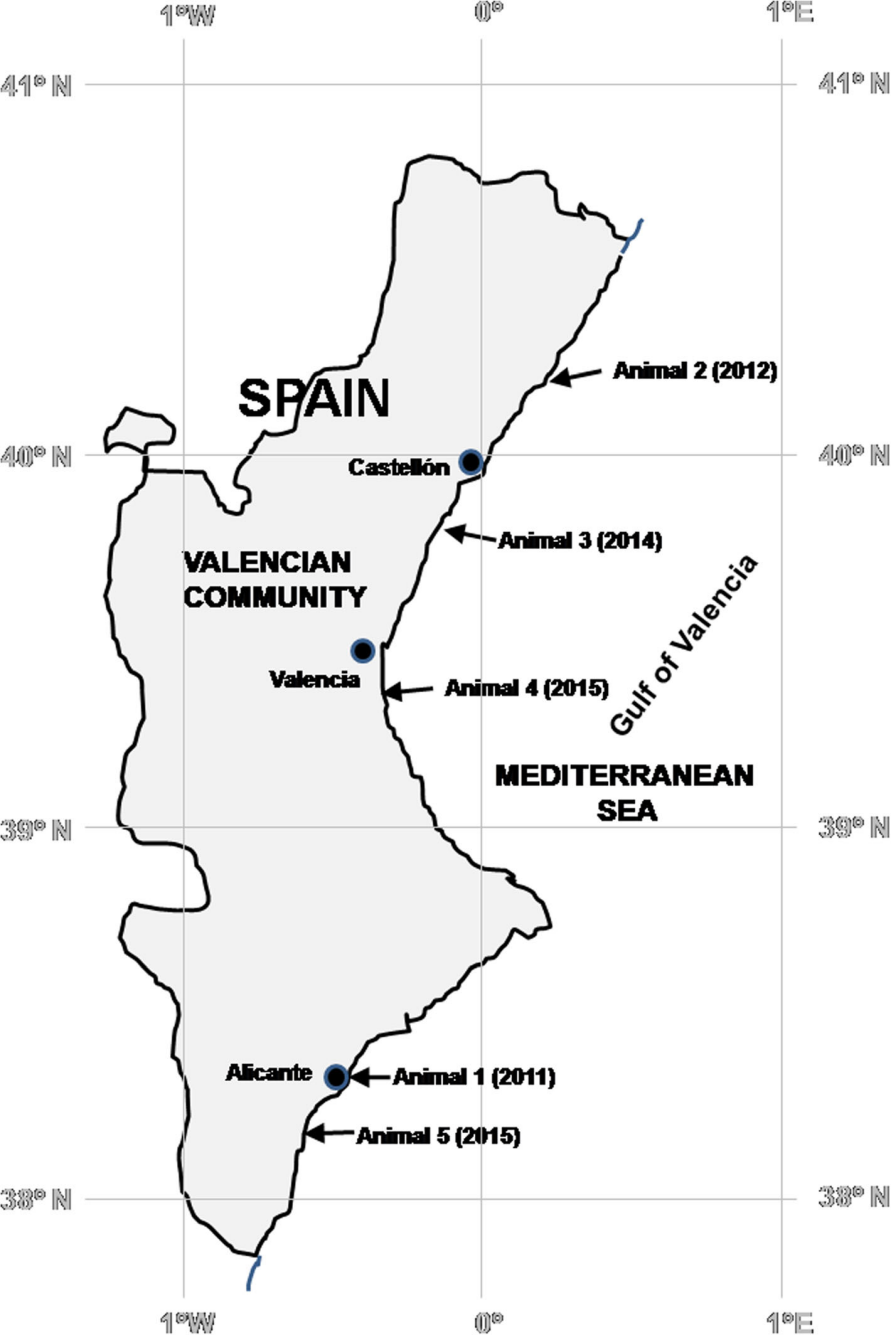
Schematic cartographic representation of the coastal area with the sites of stranding of the five DMV-positive dolphins studied here. Arrows mark the locations of the strandings, giving the identification numbers of the animals and the year of stranding (between parentheses)

Of 92 dolphins stranded on the coast of the Valencian Community of the Spanish Mediterranean that were necropsied between 2011 and 2016, only five animals, all of them striped dolphins, were DMV-positive. Of these five, animals 2, 4 and 5 stranded dead, whereas the other two animals died shortly after being found. Table 1 and Fig. 1 give the dates and locations of the strandings and relevant features of the stranded animals.

**Table 1:** Animals and stranding information

Animal #	Species	Date and place of stranding		Age Sex	Comments
1	*Stenella coeruleoalba*	July 2011	Alicante 38° 11′ 32.26” N 0° 33′ 18.75” W	Adult Female	Stranded alive 3 months after outbreak end
2	*Stenella coeruleoalba*	October 2012	Alcossebre 40° 14′ 44.75” N 0° 16′ 34.14″ E	Juvenile Male	Dead when found
3	*Stenella coeruleoalba*	July 2014	Nules 39° 50′ 41.69” N 0° 5′ 56.50” W	Adult Male	Alive with tremors
4	*Stenella coeruleoalba*	November 2015	Sueca 39° 12′ 12” N 0° 18′ 40.78” W	Adult Male	Dead when found
5	*Stenella coeruleoalba*	December 2015	Torrevieja 37° 59′ 5.00′´ N 0° 40′ 51.00” W	Calf Female	Dead when found

### Necropsy and tissue sampling

Postmortem examinations were performed within 24 h after stranding. Samples of lung, kidney, pulmonary, prescapular and mesenteric lymph nodes, pharyngeal tonsils, urinary bladder, spleen, skin, cerebellum and cerebrum were collected. All these organs, except the mesenteric lymph node and the urinary bladder, were investigated also by RT-PCR for CeMV.

### Histopathology and immunohistochemistry

Samples from virtually all organs and tissues were collected and fixed in 10% neutral buffered formalin, processed for histopathology using routine methods, and stained with hematoxylin and eosin (HE) according to standard laboratory procedures. Immunohistochemistry to detect morbilliviral antigen was done on cerebrum, lung, kidney, urinary bladder, stomach and intestine of animal 1; lung and cerebrum of animals 2 and 3; lung and pharyngeal tonsil of animal 4; and lung of animal 5. It was carried out on formalin-fixed, paraffin-embedded tissue (FFPE) sections, using IgG2B-isotype monoclonal antibody against Canine Distemper Virus nucleoprotein (CDV-NP; VMRD® Inc., USA), known to cross-react with CeMV [4, 16, 26]. Sections of FFPE canine brain with known CDV infection and immunopositivity were used as positive controls. For negative controls, the primary antibody was replaced with normal mouse serum [27].

### Molecular diagnosis and phylogenetic analysis

For DMV molecular diagnoses we used RNA isolated from homogeneized tissues (Bullet Blender™homogeneizer, Next Advance, Averill Park, NY, USA) with the NucleoSpin RNA II kit (from Macherey-Nagel, Duren, Germany), utilizing our recently described Universal Probe Library (UPL) reverse transcription PCR (RT-PCR) assay, which targets the gene for the viral fusion protein [28]. This real-time PCR assay was performed on the tissue samples routinely used to detect CeMV (described above in the section “tissue sampling”). Afterwards, portions of the viral genes for phosphoprotein (P) [29, 30], nucleoprotein (N) [31] and hemagglutinin (H) [4] were amplified in conventional RT-PCR assays using as templates pure RNA from DMV-positive lung samples from animals 1, 2, 4 and 5, and from a DMV-positive cerebrum sample from animal 3. RNA extracted from the brain of a DMV-positive striped dolphin stranded in 2007 on the Spanish Mediterranean coast served as a positive control. As a negative control, nuclease-free water was used instead of tissue-derived template.

The PCR-amplified regions of the P, N and H genes were purified with the QIAquick PCR purification kit (QIAGEN, Hilden, Germany) and Sanger-sequenced in an ABI Prism 3730 apparatus (Applied Biosystems, Foster City, CA, USA) by a commercial sequencing service (Secugen, CIB-CSIC, Madrid, Spain). These sequences were compared using BLASTN (http://blast.ncbi.nlm.nih.gov) with all the sequences of CeMV and other morbillivirus species deposited in the GenBank. Phylogenetic analysis was carried out using MEGA 4.0 software [32]. P-distance matrices were calculated and tree topology was inferred by the neighbor-joining method based on p-distances. Topology reliability was tested by bootstrapping 2000 replicates generated with a random seed. The partial P gene sequences obtained from the five striped dolphins were aligned using ClustalX to check for differences.

Diagnostic tests for *Brucella* spp. and *Toxoplasma gondii* were carried out on DNA extracted from brain homogenates from animals 1 to 4, all of which had histopathological traits of non-suppurative encephalitis because there have been some cases of combination of these infectious agents with CeMV in Italy [33, 34]. All these animals tested negative for *Brucella* spp. and *Toxoplasma gondii*. The *Brucella spp* assay was based on TaqMan Real time PCR amplification of the brucellar insertion sequence IS711 [35]. For *T. gondii*, nested PCR was used to target a sequence of the repetitive gene *B1* of this microorganism [36].

## Results

### Necropsy examination

The most relevant gross findings in animal 1 were marked prescapular and pulmonary lymphadenomegaly, pharyngeal tonsil enlargement, and multifocal pulmonary atelectasis. In Animal 2, the lungs were bilaterally expanded with noticeable rib impressions on the dorsolateral surfaces and were diffusely mottled pale gray to dark red. The meninges were diffusely congested and prescapular lymph nodes were enlarged. Animal 3 had focal ventral pulmonary atelectasis and mild caudolateral emphysema. Both prescapular lymph nodes were diffusely congested. The pulmonary-associated lymph nodes were enlarged, edematous and congested. Animal 4 had external cutaneous lesions consistent with interspecific aggressive interaction, throughout the whole body. Multiple organs and cavities were hemorrhagic because of evidence of internal lesions possibly derived from this aggressive interaction. Animal 5 was poorly preserved, and the majority of organs were severely autolyzed. However, there was clear evidence that the lungs were atelectatic and that the meninges were diffusely congested, being difficult to dissect.

### Histologic and immunohistochemical examination

Animal 1 had diffuse interstitial pneumonia with type II pneumocyte hyperplasia and syncytial cells. The spleen, prescapular, mesenteric and pulmonary-associated lymph nodes were diffusely and moderately depleted of lymphocytes and showed scattered foci of lymphocytolysis. Animal 2 also had severe multifocal bronchointerstitial pneumonia with type II pneumocyte hyperplasia and alveolar septa thickened by infiltrates of lymphocytes, plasma cells, and reactive fibroblasts. Alveoli contained macrophages and syncytial cells mixed with edema fluid. Multiple round, acidophilic intranuclear and cytoplasmic inclusion bodies were observed in syncytial cells, macrophages and type II pneumocytes. There was also evidence of multifocal lymphoplasmacytic encephalitis, with perivascular cuffing, multifocal acute neuronal necrosis, astrocytosis, and gitter cell infiltration. There was marked lymphoid depletion in the spleen and in pulmonary lymph nodes, which also presented sinus histiocytosis and syncytia. In animal 3 there was multifocal lymphoplasmacytic and histiocytic meningoencephalitis with perivascular cuffing. It also presented lymphoplasmacytic and histiocytic bronchointerstitial pneumonia with mild fibrosis, edema and a focal granuloma, and, in the examined lymph nodes, marked diffuse lymphoid depletion with sinus edema and histiocytosis. In animal 4 mixed interstitial bronchopneumonia with intralesional syncitia and both intranuclear and cytoplasmic eosinophilic inclusion bodies were observed. There was also neutrophilic and histiocytic lymphadenitis and pharyngeal tonsillitis, with syncitia and inclusion bodies. Although in animal 5 autolysis masked cellular detail preventing histological evaluation in most tissues, moderate multifocal interstitial bronchopneumonia was evidenced.

**Fig. 2:**
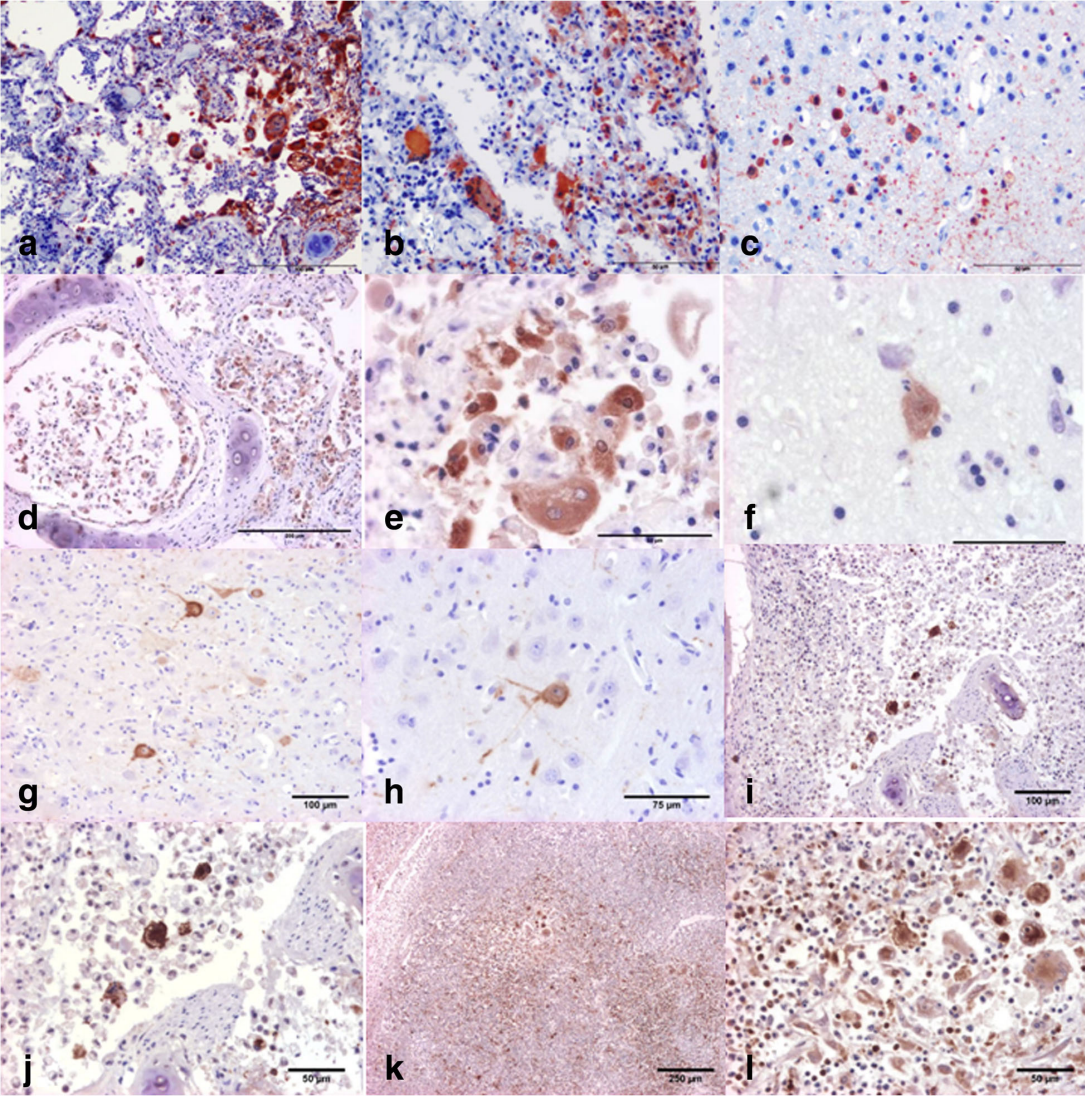
Immunohistochemistry of animals 1 (**a**-**c**), 2 (**d**-**f**), 3 (**g**-**h**) and 4 (**i**-**l**) using avidin-biotin-peroxidase and Harris hematoxylin counterstain. **a** Lung. Intense positive immunoperoxidase staining of morbilliviral antigen (red) within hyperplastic type II pneumocytes and multinucleated syncytial cells. **b** Prescapular lymph node. Positive intranuclear and intracytoplasmic immunoperoxidase staining of morbilliviral antigen in mononuclear and multinucleated giant and syncytial cells. ***c**** Cerebrum*. Strong immunolabeling of morbilliviral antigen in neurons, glial cells, and neuronal processes. **d** Lung. Intense cytoplasmic immunolabeling of alveolar syncytia, pneumocytes and sloughed bronchiolar epithelium. **e** Lung. Detail of positive cytoplasm immunolabeling of alveolar syncytia and type II pneumocytes. **f** Cerebrum. Detail of intense immunolabeling of a neuronal body. **g** Cerebrum. Strong immunolabeling of neurons (× 10). **h** Cerebrum. Detail of intense immunolabeling of a neuronal body (× 20). **i**. Lung. (× 10) and **j** Lung. (× 20). Alveolar spaces containing large numbers of histiocytes, syncytia and sloughed type II pneumocytes with positive cytoplasmic staining against morbillivirus antigen. **k** Pharyngeal tonsils (× 4) and **l** Pharyngeal tonsils (× 20). Multifocal histiocytic infiltrates and syncitya with intensely stained cytoplasm against morbillivirus antigen

The bronchointerstitial pneumonia, syncitia, inclusion bodies and encephalitis in these animals were indicative of morbilliviral infection, which was confirmed by immunohistochemistry for morbilliviral antigen. In Animal 1, the lung was moderately immunopositive (syncytial cells, type II pneumocytes, alveolar and interstitial macrophages, Fig. 2a), and there was intranuclear and cytoplasmic positivity in mononuclear and multinucleated giant and syncytial cells of the prescapular lymph node (Fig. 2b) and in brain oligodendroglia and astrocytes (Fig. 2c). Mild cytoplasmic immunopositivity was observed in the transitional epithelium of the urinary bladder and in kidney’s cortical and medullary tubular epithelium. In animal 2 immunopositivity was observed in pulmonary syncytial cells, type II pneumocytes and macrophages (Fig. 2d and e), in cerebral neurons (Fig. 2f) and gitter cells, whereas in animal 3 neuronal cell bodies and dendritic processes were intensely immunoreactive (Fig. 2g and h), with no immunoreactivity detected in lung tissue. In animal 4 pulmonary pneumocytes and syncitia and histiocytes of lung (Fig. 2i and j) and pharyngeal tonsils (Fig. 2k and l) were strongly labelled. The lung from animal 5 was clearly immunopositive for morbilliviral antigen.

### Molecular and phylogenetic analyses

As expected for systemic morbilllivirus infection, all tissues tested from animals 1, 2, 4 and 5 were strongly positive to the fusion protein gene by RT-PCR (assayed by UPL RT-PCR) (Table 2) and for the P, N and H genes (assayed in conventional PCR assays). In contrast, in animal 3 only CNS samples were strongly positive for mobillivirus by these same criteria, whereas all other tissues proved negative for these four genes (Table 2), indicating CNS-restricted infection in this animal.

**Table 2:** Samples analyzed and results obtained in the molecular and immunohistochemical studies of our animals^a^

Animal	Conventional RT-PCR for CeMV	UPL RT-PCR for CeMV	Immunohistochemistry for CDV
1	Skin, lung, prescapular lymph node, pulmonary lymph node, kidney, pharyngeal tonsils, cerebrum, cerebellum	Skin, lung, prescapular lymph node, pulmonary lymph node, kidney, pharyngeal tonsils, cerebrum, cerebellum	Lung, cerebrum, kidney, *intestine, stomach*, urinary bladder
2	Skin, lung, prescapular lymph node, pulmonary lymph node, kidney, pharyngeal tonsils, cerebrum, cerebellum	Skin, lung, prescapular lymph node, pulmonary lymph node, kidney, pharyngeal tonsils, cerebrum, cerebellum	Cerebrum, lung
3	*Skin, lung, prescapular lymph node, pulmonary lymph node, kidney, pharyngeal tonsils,* cerebrum, cerebellum	*Skin, lung, prescapular lymph node, pulmonary lymph node, kidney, pharyngeal tonsils,* cerebrum, cerebellum	*Lung*, cerebrum
4	Skin, lung, prescapular lymph node, pulmonary lymph node, kidney, pharyngeal tonsils, cerebrum, cerebellum	Skin, lung, prescapular lymph node, pulmonary lymph node, kidney, pharyngeal tonsils, cerebrum, cerebellum	Lung, pharyngeal tonsils
5	Skin, lung, prescapular lymph node, pulmonary lymph node, kidney, pharyngeal tonsils, cerebrum, cerebellum	Skin, lung, prescapular lymph node, pulmonary lymph node, kidney, pharyngeal tonsils, cerebrum, cerebellum	*Cerebrum*, lung

^a^The indicated tissues or organs were those analyzed. Italic type highlights tissues that tested negative

**Fig. 3:**
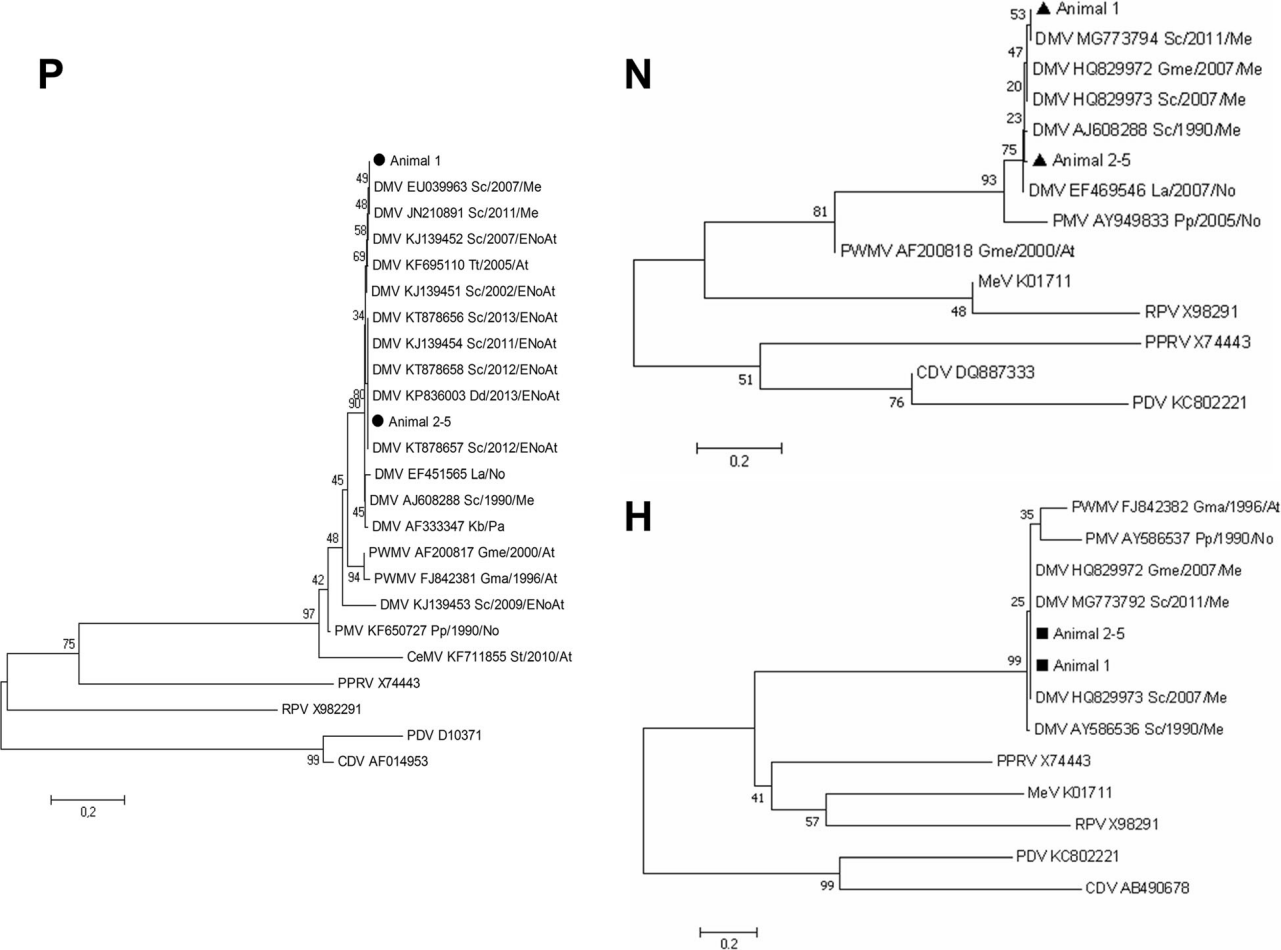
Phylogenetic analysis. Neighbor-joining phylogram of morbillivirus phosphoprotein (P), nucleoprotein (N), and hemagglutinin (H) gene sequences. The name of each sequence is composed of the virus name (CeMV, cetacean morbillivirus; DMV, dolphin morbillivirus; PMV, porpoise morbillivirus; PWMV, pilot whale morbillivirus; PPRV, peste-des-petits-ruminants virus; RPV; rinderpest virus; CDV, canine distemper virus; PDV, phocine distemper virus; MeV, measles virus), the GenBank accession number, the infected cetacean species (Gma, *Globicephala macrorhynchus;* Gme*, Globicephala melas;* Kb, *Kogia breviceps;* La, *Lagenorhynchus australis;* Pp, *Phocoena phocoena;* Sc, *Stenella coeruleoalba;* St, *Sotalia guianensis*; Tt, *Tursiops truncatus*), and the year and geographic area of stranding (At, Atlantic Ocean; ENoAt, Western Atlantic; Me, Mediterranean Sea; No, North Sea; Pa, Pacific). “Animal 1” and “Animal 2–5” indicate the sequences obtained from cerebrum samples from the dolphins in the present study. The new isolates from this study have the GenBank accession numbers KC572861 (P gene), MG773795 (N gene) and MG773793 (H gene)

**Fig. 4:**
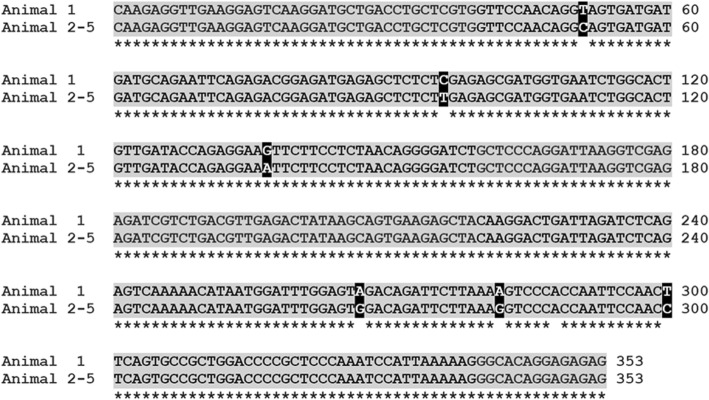
Alignment of partial P gene sequences from animal 1 and from animals 2–5. Identical regions are shadowed gray and marked with asterisks at the bottom. Differences are highlighted using black shadowing over white lettering

Subsequent Sanger sequencing confirmed that all amplicons were mobilliviral sequences. The nucleotide sequences from the P gene, N gene and H gene of animal 1 corresponded to those reported in animals of the 2011 Spanish Mediterranean outbreak (P gene, GenBank JN210891; N gene MG773794; H gene, MG773792), indicating that the DMV responsible for that outbreak caused the systemic infection of animal 1. However, the P, N and H sequences derived from animals 2 to 5 differed from those of animal 1, being identical in these four animals (P gene, GenBank KC572861; N gene, GenBank MG773795; H gene, MG773793) (Figs. 3 and 4).

## Discussion

The results of this study show that five striped dolphins stranded in the Spanish Mediterranean between 2011 and 2015 had an infectious disease presenting with variable degrees of bronchointerstitial pneumonia, non-suppurative encephalitis and multicentric lymphoid depletion. In animals 1, 2 and 4, which had good tissue preservation, the pathological observations in several organs (Fig. 2) were consistent with acute systemic morbilliviral infection, with immunohistochemical confirmation. In addition, molecular tests proved the presence of DMV in multiple tissues of these animals and of the poorly preserved animal 5 (Table 2). In this last animal, lung preservation was sufficient to also reveal typical mobilliviral pathology and immunohistochemistry (Fig. 2). These animals are the first reported cases in the Spanish Mediterranean Sea of acute systemic DMV infection between epizootic outbreaks. In contrast to the other four dolphins, in animal 3, which presented meningoencephalitis, the immunohistochemistry for the viral antigen and the molecular tests for DMV were only positive in cerebral tissue (Table 2). Although this animal also had bronchointerstitial pneumonia, the signs of chronicity for this derrangement, together with the negativity of the lung for the laboratory tests for DMV did not support active pulmonary disease due to this virus. At stranding, the DMV infection appears limited in this animal to the CNS, corresponding to the chronic encephalitic form of DMV infection [37].

Comparisons of viral sequences suggest that the same DMV strain infected animals 2–5, and that this strain differs from the one in animal 1, as well as from the ones detected in the Spanish Mediterranean outbreaks of 1990, 2007 and 2011 (Fig. 3) [2, 15, 16]. Instead, the strain infecting animals 2–5 appears to be identical (p-distance, 0.000) to a strain identified in the Atlantic Ocean in dolphins stranded in 2011–2013, one in the Canary Islands (GenBank KF695110) and six in the Northwestern coast of the Iberian Peninsula (GenBank, KP836003, KT878656, KT878657, KT878658, KT878660, KT878661). Thus, it appears that the DMV virus infecting animals 2–5 had an Atlantic origin [23, 25]. This conclusion is supported also by the observation that the P gene sequence of animals 2–5 is phylogenetically very close to the P gene sequence of the DMV responsible for the 1990 Spanish Mediterranean outbreak (GenBank AJ608288; p-distance, 0.012), known to have Atlantic origin [11]. This close relation was also found (Fig. 3) for the N gene sequence (p-distance 0.015) (GenBank AJ608288). P-distances for the H gene (1990; GenBank AY586536) also support the Atlantic origin of the strain found in animals 2–5. Unfortunately, the lack of reporting of these sequences for Atlantic DMV isolates prevented similar comparisons with the viruses of the 2011–2013 strandings in the Canary Islands and the Nothwestern Iberian coast.

Concerning animal 1, since it was found to contain the same DMV strain that caused the 2011 outbreak and it stranded only three months after that outbreak was declared terminated [16], it cannot be excluded that it belongs to the trailing edge of the 2011 outbreak. However, systemic cases were found in Italy in 2011 to 2013 in one fin whale and in numerous sperm whales due to DMV strains closely related to the viruses responsible for the 2007 and 2011 Spanish outbreaks [22, 24], and DMV-positive striped dolphin mass mortality also occurred in Italy in 2013 that could possibly be related to these previous Mediterranean strains [21]. Thus, as already proposed [3, 16] there appears to be endemic viral circulation within the Mediterranean, possibly within the abundant striped dolphin population, although the potential roles of whales in the transmission and maintenance of DMV strains, possible in principle given the reports of pilot whales and a Cuvier’s beaked whale (*Ziphius cavirrostris*) infected with DMV [6, 20, 38], appear unlikely given the low density of these species in the Mediterranean. The fact that the Valencian Community was the epicenter of the previous three DMV epizootics in the Mediterranean makes conceivable that this region could harbor local DMV reservoirs, although this possibility requires further investigation, since other factors like abundance of striped dolphins in the area, and efficient and active surveillance networks may be responsible for the early detection of the epizootics in this region.

Even though most cases of DMV infection outside outbreak periods appear to be of the chronic encephalitic form [30, 39], our findings highlight the need to be prepared to deal with possible cases of systemic infection within these inter-epizootic periods. In addition, our finding of sequences of apparent Atlantic origin in four of the animals dealt with here stress the importance of sequencing standard fragments of viral genes to make inferences that could suggest paths of virus circulation and provide epidemiological insight, even giving clues for early detection of impending outbreaks.

Comparisons of viral sequences from the three previous Spanish Mediterranean outbreaks suggest that the outbreaks were caused by closely related DMV strains [6, 16]. For example, our results indicate a close relationship between sequences linked to the Spanish outbreaks of 2007 and 2011. Sequence comparisons extending over 9.050 kb [6] suggest that the Spanish 2007 DMV strain is derived from the Spanish 1990 DMV strain. Our finding that the sequences from animal 1 are more closely related to the 2011 sequence (P gene, GenBank JN210891; N gene, GenBank MG773794; H gene, GenBank MG773792) than to the sequences from 1990 or 2007 could be consistent with a sequential lineage (1990 → 2007 → 2011 → DMV in animal 1) or with reintroduction from the Atlantic in 2007. In contrast to the case of animal 1, partial P, N and H gene sequences from animals 2, 3, 4 and 5 were identical to each other and appear to cluster separately from the sequences from the 1990, 2007 and 2011 isolates. Our finding that animal 2, which stranded in 2012, contained an identical P sequence to that of DMV identified in the Atlantic in a dolphin that stranded in 2011 [25], suggests an Atlantic origin for this DMV strain, which was subsequently found also in animals 3, 4 and 5. Consistent with this is the fact that animal 2 had acute disseminated disease, with the virus detected in the lung and the nervous system, which would be expected if the Mediterranean striped dolphin population in 2012 was naïve to this Atlantic strain. The fact that we found the same strain in animal 3 that stranded in 2014 with CNS-restricted infection indicates that this strain can induce both systemic and chronic infection.

## Conclusions

In summary, our results suggest that multiple DMV strains are circulating in striped dolphins and causing systemic infection in the western Mediterranean during inter-epizootic periods. At least one of these strains can produce both systemic and chronic infection and appears to have originated in the Atlantic Ocean. These findings warrant further studies aiming at clarifying how the virus circulates and causes epidemics in the Mediterranean Sea.

## Abbreviations

CDV-NP: Canine Distemper Virus nucleoprotein
CeMV: Cetacean morbillivirus
CNS: Central nervous system
DMV: Dolphin morbillivirus
FFPE: Formalin-fixed, paraffin-embedded
H: Haemagluttinin
HE: Hematoxylin and eosin
MI: Morbillivirus infection
N: Nucleoprotein
P: Phosphoprotein
PMV: Porpoise morbillivirus
PWMV: Pilot whale morbillivirus
RT-PCR: Reverse transcription PCR
UPL: Universal probe library
